# What and how do different stakeholders contribute to intervention development? A mixed methods study.

**DOI:** 10.12688/hrbopenres.13544.1

**Published:** 2022-05-10

**Authors:** Emmy Racine, Lauren O Mahony, Fiona Riordan, Gráinne Flynn, Patricia M. Kearney, Sheena M. McHugh

**Affiliations:** 1 School of Public Health, University College Cork, Cork, T12 K8AF, Ireland; 2 PPI Contributor, IDEAs Research Project, University College Cork, Cork, T12 K8AF, Ireland

**Keywords:** Intervention development, user involvement, patient and public involvement, Study Within A Trial, Diabetic Retinopathy Screening.

## Abstract

**Background: **UK Medical Research Council guidelines recommend end-user involvement in intervention development. There is limited evidence on the contributions of different end-users to this process. The aim of this Study Within A Trial (SWAT) was to identify and compare contributions from two groups of end-users - people with diabetes’ (PWD) and healthcare professionals’ (HCPs), during consensus meetings to inform an intervention to improve retinopathy screening uptake.

**Methods:** A mixed method, explanatory sequential design comprising a survey and three semi-structured consensus meetings was used. PWD were randomly assigned to a diabetes only or combined meeting. HCPs attended a HCP only or combined meeting, based on availability. In the survey, participants rated intervention proposals on acceptability and feasibility. Survey results informed the meeting topic guide. Transcripts were analysed deductively to compare feedback on intervention proposals, suggestions for new content, and contributions to the final intervention.

**Results:** Overall, 13 PWD and 17 HCPs completed the survey, and 16 PWD and 15 HCPs attended meetings. For 31 of the 39 intervention proposals in the survey, there were differences (≥10%) between the proportion of HCPs and PWD who rated proposals as acceptable and/or feasible. End-user groups shared and unique concerns about proposals; both were concerned about informing but not scaring people when communicating risk, while concerns about resources were mostly unique to HCPs and concerns about privacy were mostly unique to PWD.  Fewer suggestions for new intervention content from the combined meeting were integrated into the final intervention as they were not feasible for implementation in general practice. Participants contributed four new behaviour change techniques not present in the original proposals: *goal setting (outcome)*, *restructuring the physical environment*, *material incentive (behaviour)* and *punishment*.

**Conclusions:** Preferences for intervention content may differ across end-user groups, with feedback varying depending on whether end-users are involved simultaneously or separately.

## List of abbreviations

**APEASE**    Affordability, Practicability, Effectiveness, Acceptability, Side effects and Equity

**BCT**            Behavioural Change techniques

**DRS**            Diabetic Retinopathy Screening

**GP**              General Practitioner

**HCP**           Health Care Professional

**NHS**           National Health Service

**PN**              Practice Nurse

**PPI**             Patient and Public Involvement

**PWD**          People With Diabetes

**SMS**           Short Message Service

**SPSS**          Statistical Package for the Social Sciences

**SREC**         Social Research Ethics Committee

**SWAT**         Study Within A Trial

## Introduction

According to the UK Medical Research Council guidance on the development and evaluation of complex interventions, interventions should be developed with user involvement, drawing on existing evidence and appropriate theory ^1^. User involvement usually includes those who will deliver the intervention (often healthcare professionals [HCPs]) and the intended target population (often patients and the public). It is expected to improve the intervention fit with the target group’s perceived needs enhancing acceptability; feasibility; evaluability and adoption ^2, 3^.

While some studies have found that different end-users have similar priorities and preferences when making decisions about health research and service delivery ^4, 5^, other studies have found that different end-users endorse different perspectives ^6, 7^. In the context of intervention development, limited evidence exists on what different intervention users contribute to the process. Morton *et al*. have suggested that different stakeholders may have different priorities for intervention content ^8^. For instance, the cost of a proposed intervention might be more important than feasibility for intervention commissioners, whereas those receiving the intervention may be more concerned with its acceptability. However, more substantive research is needed to empirically examine and compare what different end-users contribute to the intervention development process. 

Furthermore, group dynamics are complex, and some user groups may find it more difficult to voice their priorities and perspectives compared with others ^9^. Studies involving end-users in intervention development tend to treat all end-users (e.g., patients and HCPs) as one homogenous group ^10– 12^. We previously compared participants’ experiences of taking part in meetings to inform the development of an intervention to increase diabetic retinopathy screening attendance ^13^. Three meetings were held comprising people with diabetes only; a combined meeting of people with diabetes and HCPs; and a HCP only meeting. We found that involving both people with diabetes and HCPs in the same group led to a perceived lack of common ground where both groups felt undervalued by the other group and were reluctant to express their opinions ^13^. While these findings might suggest that intervention end-users may find it more acceptable to involve each group separately, we are also keen to know whether their contributions during these meetings differed according to group composition. Understanding whether user contributions differ according to group composition could enable researchers to design and conduct more appropriate and effective user involvement activities which in turn could potentially improve intervention fit with the target group’s perceived needs. 

The aim of this Study Within A Trial (SWAT) was to identify and compare people with diabetes’ and HCPs’ contributions during three consensus meetings to inform intervention development, including their feedback on the acceptability and feasibility of intervention content, suggestions for new intervention content, and contributions to the final intervention.

## Methods

This SWAT was embedded in the intervention development phase of the Improving Diabetes Eye-Screening Attendance (IDEAs) pilot trial ^14^. IDEAs used a systematic three-step process combining theory, user involvement and evidence on intervention effectiveness to develop a multifaceted intervention targeting people with diabetes and HCPs to improve uptake of RetinaScreen, a national Diabetic Retinopathy Screening (DRS) programme ^15^. As part of the user involvement process, three semi-structured consensus meetings were conducted to review and discuss proposals for intervention content.

### Design

This SWAT is a mixed method study using an explanatory sequential design ^16^. Quantitative data (self-completion participant survey) were collected and analysed first, followed by the qualitative data (consensus group meetings) which were collected and analysed second in sequence ^17^. The quantitative results provided an overview of participant ratings of acceptable and feasible intervention content, while the qualitative analysis allowed for further exploration of why participants rated intervention content the way they did by using a topic guide informed by survey findings.

### Recruitment

***People with diabetes***

People with diabetes were recruited using an information flyer developed by the research team including a graphic designer ( http://doi.org/10.5281/zenodo.4321202). The flyer was distributed using a range of recruitment strategies including social marketing recruitment, community outreach recruitment, health system recruitment, and partnering with other organisations. All individuals who contacted the study team and returned a short demographic survey (Supplementary File 1 in the *Extended data* ^18^) were randomly assigned (using an online random number generator) to either the meeting for the people with diabetes only, or the combined meeting.

***Health care professionals***

HCPs were recruited through local professional networks known to the study team. An email invitation was sent to 50 HCPs (practice nurses, diabetes nurse specialists, general practitioners, and specialist physicians). All HCPs were allocated based on their availability to the HCP-only meeting or combined meeting. Further details on the recruitment process have been described in detail elsewhere ^13^.

### Data collection

***Quantitative phase***

Before each consensus meeting, participants were sent an evidence summary of barriers to and enablers of attendance at diabetic retinopathy screening, and interventions to address non-attendance (Supplementary File 2 in the *Extended data* ^18^), and a self-completion survey (Supplementary File 3 in the *Extended data* ^18^). The evidence summary and survey were designed with input from the Irish National Adult Literacy Agency and a Patient and Public Involvement (PPI) group and revised based on their feedback.

The survey outlined 39 proposals for intervention content that were grouped at the practice-level (‘ways to encourage the practice staff to make sure person attends’) and patient-level (‘ways to encourage the person to attend diabetes eye screening’). The proposals contained operationalised behaviour change techniques (BCTs), defined as an “observable, replicable, and irreducible components of an intervention” that have the potential to change behaviour ^19^. The proposals (operationalised techniques) were short statements/descriptions of how the selected BCT would be put into practice ^20^, in line with the study focus on increasing diabetic retinopathy screening uptake. The BCTs in the survey were selected to address known barriers to and enablers of screening attendance based on previous formative research conducted by the IDEAs research team ^15^ and existing evidence of their effectiveness either in interventions to increase retinopathy screening attendance or interventions in other settings ^21, 22^. A total of 24 unique BCTs were operationalised across the 39 intervention proposals in the survey. Further details on these 24 BCTs has been provided in Supplementary File 4 in the *Extended data* ^18^.

In the survey, participants were asked to rate the acceptability and feasibility of each proposal. All items were rated on a Likert response scale ranging from 1 to 5 (from ‘strongly disagree’ to ‘strongly agree’) with higher scores indicating greater acceptability or feasibility. These survey questions were adapted from existing measures developed by Weiner *et al.* to rate implementation acceptability and feasibility ^23^. Acceptability was defined as the perception among end-users that the intervention proposal was agreeable or satisfactory. Feasibility was defined as the extent to which the intervention proposal could be successfully implemented in general practice. People with diabetes received a paper format of the survey while HCPs received an electronic format. 

***Qualitative phase***

Following completion of the surveys, participants took part in one of three consensus group meetings. Each meeting was held for two hours in University College Cork and was facilitated by the same facilitator experienced in consensus group techniques/processes. This facilitator was a male professor of health services research who held no relationship with participants.

During the meetings, a summary of the ratings of acceptability/feasibility was presented to participants. This was followed by a series of small group discussions (facilitated by members of the research team) where participants were asked to discuss how each intervention proposal would work in practice (See Supplementary File 5 in the *Extended data* ^18^ for Facilitator Guide). Facilitators asked participants to discuss and give feedback on both practice-level and patient-level proposals. Prompts about patient-level proposals included 1) who should deliver the message to remind patients to attend diabetes eye screening? 2) how should the message be delivered? 3) when should the message be delivered? and 4) what should the message contain? Participants were asked to focus their discussion on proposals where the consensus on acceptability and feasibility based on the survey was unclear. However, given the semi-structured nature of the meetings, participants also made new suggestions. The small group discussions and the feedback to the larger group were digitally audio recorded with participant consent.

### Data analysis

Participant survey responses were entered into SPSS software (version 26, RRID:SCR_016479) and analysed using descriptive statistics. Consensus meeting transcripts were analysed using NVivo 12 software (RRID:SCR_014802). If this software were unavailable, it would be possible to conduct the analysis using Excel and Word.

***Comparing end-users’ feedback on the acceptability and feasibility of intervention content***

To examine participants’ ratings of the acceptability and feasibility of intervention proposals, the five-point Likert scale used in the survey was collapsed into three categories: ‘disagree’ [1 strongly disagree, 2 disagree], ‘neither disagree or agree’ [3] and ‘agree’ [4 agree, 5 strongly agree]. Contingency tables were generated for each intervention proposal by participant type (HCP or people with diabetes) and Fisher’s exact test was used as appropriate ^24^. Results were examined to identify proposals which had a difference (≥10%) between the proportion of HCPs and people with diabetes who agreed that intervention proposal was feasible and/or acceptable.

Guided by the survey results, interview transcripts were analysed using deductive content analysis. A codebook (developed *a priori* by LOM) designed to mirror the self-completion survey to identify and code feedback on specific proposals was used. Participants in the combined meeting were asked to reach group consensus on intervention proposals, therefore it was difficult to attribute feedback exclusively to people with diabetes or HCPs or both. Therefore, the people with diabetes only meeting and the HCP only meeting were analysed *before* the combined meeting was analysed, to allow the researchers to see whether feedback from the combined meeting echoed that of the people with diabetes only and HCP only meetings.

**Figure 1.**
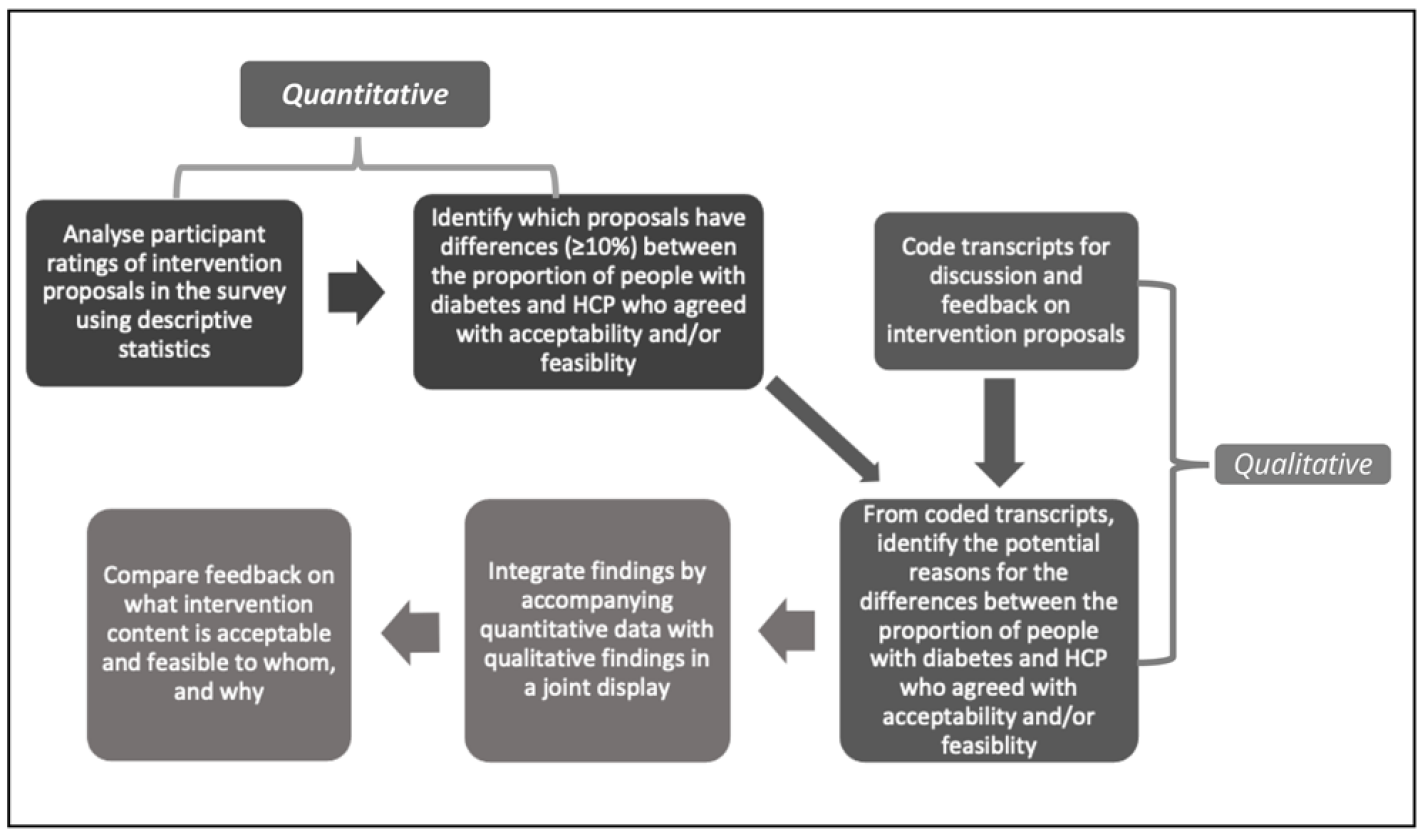
Overarching sequence of mixed methods.

To compare participants’ feedback on the acceptability and feasibility of intervention proposals, thematic analysis was performed by LOM, guided by joint displays of the survey results and qualitative coding. The joint displays were examined for recurring patterns between survey ratings and discussion during the consensus meetings, to identify reasons for agreement/disagreement e.g., what was or was not acceptable/feasible to whom, and why. An overview of this sequence of mixed methods is provided in Figure 1.

***Comparing end users’ suggestions for new intervention content***

To identify and compare end-users’ suggestions for new intervention content, two researchers (ER and FR) conducted a deductive content analysis ^25^ to identify suggested changes to proposed intervention content and suggestions of additional intervention content. Both researchers read the consensus meeting transcripts multiple times (data familiarisation) and then independently extracted all suggestions made by participants in relation to intervention content and mode of delivery. A suggestion was defined prior to data analysis as any suggestion about intervention content or mode of delivery proposed by a member of the group, at any stage during the meeting, that was agreed with by one or more other members of the group. Agreement or disagreement between participants was ascertained based on explicit verbal expression or sounds or noises which conveyed their agreement or disagreement (e.g., mmm). The two researchers met to discuss the suggestions they had extracted. Any differences were discussed, and agreement was reached by consensus on the list of suggestions put forward by participants. Each new suggestion was then coded (yes/no) according to whether it would be feasible to incorporate into the intervention to be delivered. The scope of the intervention was defined as:

- purpose of the intervention (to improve the uptake of a national DRS service)
- intervention setting (general practice in Ireland)
- timeline (2 years to develop and test the feasibility of the intervention)
- budget (the IDEAs study was providing a practice participation fees plus some materials/consumables approx. €1,000 per practice)
- practice resources (each practice needed to have at least one practice nurse and computerised patient records)

To identify how each new suggestion aligned with existing behavioural change techniques, they were mapped to Behaviour Change Technique Taxonomy (BCTTv1) ^26^. Further information on how this mapping was conducted is provided in Supplementary File 6 in the *Extended data* ^18^.

***Comparing end users’ contributions to the final intervention***

Using deductive content analysis, one researcher (ER) categorised (yes/no) all recommendations (including feedback on proposals and suggestions for new intervention content) according to whether they were incorporated into the final intervention. Full details about the decision process regarding the final intervention content has been published elsewhere ^15^. The final decision on the intervention content was made by a subgroup of the IDEAs study research team and a GP collaborator, basing decisions on the APEASE (affordability, practicability, effectiveness, acceptability, side effects, equity, sustainability) criteria. Practicality and acceptability criteria were populated based on findings from the rating survey and the discussions during the consensus meetings. The effectiveness criterion was based on a rapid evidence review of different approaches to improve screening uptake. Remaining criteria (affordability, equity, side-effects (unintended consequences), sustainability) were based on group discussions about what was feasible, bearing in mind previous formative research with patients and healthcare professionals and organisational factors relating to the primary care environment.

### Patient and Public Involvement (PPI)

A PPI contributor (GF) was involved in the SWAT from the outset. GF is a person with diabetes, previously known to the lead author (ER). She contributed to the initial discussions about the study which ultimately informed the SWAT grant application, reviewed the grant application prior to submission and made changes to its content including the addition of disseminating the research amongst people with diabetes. GF was also involved in the development of materials used to recruit people with diabetes and assisted the research team with recruitment by posting recruitment flyers online via social media networks. She contributed to and reviewed each draft of this manuscript and is a co-author on this publication. The IDEAs study worked with a dedicated PPI group throughout the duration of the trial. Before the IDEAs PPI group was established, the lead author worked with a separate primary care research PPI group to develop and refine the materials that were sent to participants prior to the consensus meeting.

### Ethical approval

The study received ethical approval from the Social Research Ethics Committee (SREC) at University College Cork (Log number 2018-122, approval received 13/08/2018). Written informed consent was obtained from all participants prior to completing the rating survey and taking part in the consensus meetings.

## Results

### Comparing end users’ feedback on the acceptability and feasibility of intervention content

In total, 30 participants (13 people with diabetes and 17 HCPs) completed and returned the surveys. Missingness within the data ranged from 3.3% to 6.7%, depending on the survey proposal. There was incomplete data for 6 participants (4 people with diabetes, 2 HCPs). Table 1 presents the 31 proposals which had differences (≥10%) between the proportion of HCPs and people with diabetes who agreed the proposal was acceptable and/or feasible ^18^.

**Table 1.** Patient-level and practice-level proposals with survey results and related concerns identified from the consensus meetings.

Intervention Component ( *embedded BCT*)	Proposal ( *Operationalised BCT*)	Self-completion survey						Semi-structured consensus meetings
		Agreed proposal acceptable			Agreed proposal feasible			Related concern *(or* * preference)* where indicated in the data (Joint; HCP; People with diabetes)
		People with diabetes % (n)	HCP % (n)	Diff. %	People with diabetes % (n)	HCP % (n)	Diff. %	
**Patient-level proposals**								
**(i) Using a personal story from** **someone else with diabetes** **who delivers the message** **who…** **( *9.1 Credible source*)**	…is a similar age and profile to People with diabetes and explains how screening was a way for them to take charge of their health. *(6.2 Social comparison and 15.1 Verbal persuasion* *about capability)*	84 (11)	64.7 (11)	19.3	75 (9)	58.8 (10)	16.2	NA
	…has retinopathy and tells them the benefits of screening. *(5.3 Information about social and environmental * *consequences and 11.2 reduce negative emotions)*	92.3(12)	82.2 (15)	10.1	100 (12)	58 (10)	42	Balancing act: informing but not scaring people with diabetes (Joint)
	…has retinopathy and tells them it is important to go to screening before it is too late, there may be no symptoms and everyone with diabetes is at risk. *(5.1 information about health consequences and* * 5.5 Anticipated regret)*	100 (13)	94.1 (16)	5.9	83.3 (10)	64.7 (11)	18.6	
	…wishes they went to screening sooner and prompts the person to think about the regret they will feel if they do not attend screening. *(5.5 Anticipated regret and 6.2 Social comparison)*	84.6 (11)	58.8 (10)	15.8	75 (9)	47.1 (8)	27.9	
	…explains there is no harm from drops used during screening and the overall benefits outweigh the short-term discomfort. *(5.1 Information about health consequences)*	92.3 (12)	82.4 (14)	9.9	83.3 (10)	64.7 (11)	18.6	NA
	…provides an observable example that shows them how to consent or attend. *(6.1 Demonstration of behaviour)*	76.9 (10)	76.5 (12)	0.4	75 (9)	52.9 (9)	22.1	NA
	…delivers a message recognising the anxiety people might feel but emphasizes the positive consequences of attending. *(11.2 Reducing negative emotions)*	100 (13)	94.1 (16)	5.9	83.3 (10)	64.7 (11)	18.6	NA
	…prompts the person to imagine the outcomes of attending vs. not attending. *(9.2 Pros and cons)*	84.6 (11)	52.9 (9)	31.7	66.7 (8)	47.1 (8)	19.6	NA
**(ii) Someone in the practice ** **could…** ***(9.1 Credible source)***	…Encourage the person to attend screening. *(3.1 Social support (unspecified))*	92.3 (12)	100 (17)	7.7	75 (9)	94 (16)	19	NA
	…Tell the person that they approve of screening and hope the person will attend. *(6.3 Information about other’s approval)*	92.3 (12)	94.1 (16)	1.8	83.3 (10)	93.8 (15)	10.5	NA
	…Persuade the person they will be able to attend screening (e.g., help them to think about times they successfully managed their diabetes or attended appointments). *(15.3 Focus on past success)*	69.2 (9)	76.5 (13)	7.3	58.3 (7)	70.6 (12)	12.3	NA
	…Explain the difference between routine eye checks and the screening test, what both tests can and cannot tell them, and that routine checks are not a substitute. *(5.1 Information about health consequences)*	92.3 (12)	94.1 (16)	1.8	75 (9)	94.1 (16)	19.1	*Some people with diabetes* * have a limited understanding* * of the need for and* * practicalities of screening * *(Joint preference)*
	…Advise the person how to consent to screening and to ask for help if they are unable/unsure about how to do this *(4.1 Instruction on how to perform the behaviour* * and 3.2 Social support (practical))*	92.3 (12)	100 (17)	7.7	91.7 (11)	76.5 (13)	15.2	NA
	…Tell the person that after their appointment they will be reassured, or they can get treated in time to stop things getting worse. *(5.1 Information about health consequences and* * 5.6 Information about emotional consequences)*	100 (13)	94.1 (16)	5.9	91 (11)	76.5 (13)	14.5	NA
	…Explain how it’s important to go to screening before it is too late, they personally are at risk and that screening applies to them. *(5.1 Information about health consequences* * and 5.5 Anticipated regret and 5.2 Salience of * *consequences)*	100 (13)	88.2 (15)	11.8	91.7 (13)	82.3 (14)	9.4	Balancing act: informing but not scaring people with diabetes (Joint)
	…Encourage the person to think of screening not as something extra, but as part of the whole package of self-management. *(13.2 Framing/reframing)*	100 (12)	88.2 (15)	11.8	90.9 (10)	88.2 (15)	2.7	
	…Help the person to make a plan about when and where they will consent and how they will attend when they get their appointment. *(1.4 Action planning)*	61.5 (8)	64.7 (11)	3.2	50 (6)	70.6 (12)	20.6	NA
**(iii) Other ideas of how to** **encourage the person to** **attend**	Arrange for support from family/friends (e.g., encouragement to consent/attend). *(3.1 Social support (unspecified))*	69.2 (9)	58.8 (10)	10.4	41.7 (5)	47.1 (8)	5.4	Risks patient privacy (People with diabetes Strays outside HCPs area of responsibility (HCPs) *Some people with diabetes* *have a limited understanding* *of the need for and* *practicalities of screening* *(Joint preference)*
	Advise/arrange for practical support like transportation from family/friends. *(3.2 Social support (practical))*	66.7 (8)	82.4 (14)	15.7	58.3 (7)	41.2 (7)	17.1	
	Draw the person’s attention to the number of people like them who have attended. *(6.2 Social comparison)*	53.8 (7)	58.8 (10)	5	50 (6)	64.7 (11)	14.7	NA
	The person with diabetes ticks off a checklist when they have consented/attended. *(2.3 Self-monitoring of behaviour)*	69.2 (9)	35.3 (6)	33.9	75 (9)	35.3 (6)	39.7	Relying on active participation from people with diabetes (Joint)
Practice-level								
**(iv) Ways to encourage the** **practice staff to make sure the** **person attends**	Provide practice with observable example/ information on how to check and register people with diabetes. *(4.1 Instruction on how to perform the behaviour* *and 6.1 Demonstration of the behaviour)*	100 (13)	94.1 (16)	5.9	91.7 (11)	76.5 (13 )	15.2	Resource implications (HCPs)
	Prompt practice to check the register during consultation and register person if necessary *(7.1 Prompts and cues)*	92.3 (12)	82.4 (14)	9.9	91.7 (11)	70.6 (12)	21.1	Resource implications (HCPs)
	Provide a new resource to the practice (e.g., researcher checks if person registered, consented and/or attended) *(12.2 Restructuring the social environment)*	83.3 (10)	64.7 (11)	18.6	53.8 (7)	58.8 (10)	5	Resource implications (HCPs) Risks patient privacy (People with diabetes)
	Provide checklist of ways to encourage consent/ attendance *(12.5 Adding objects to the environment)*	76.9 (10)	52.9 (9)	24	58.3 (7)	64.7 (11)	6.4	Resource implications (HCPs)
**(v) Telling practices about** **the benefits/consequences of** **their patients attending/not** **attending**	The benefits to the practice when their patients attend (e.g., receiving timely results, they have access to local service) *(5.3 Information about social and environmental* *consequences)*	81.8 (9)	70.6 (12)	11.2	83.3 (10)	70.6 (12)	12.7	Motivating practice staff to make sure the person attends screening (HCPs)
	Consequences when their patients do not attend (e.g., eye damage, costs of missed appointments). *(5.3 Information about social and environmental* *consequences)*	66.7 (8)	76.5 (13)	9.8	90.9 (10)	70.6 (12)	20.3	
**(vi) Use a personal story from** **a patient to inform practices…** ***(9.1 Credible Source)***	… about the benefits and risks to patients of attending/not attending *(5.1 Information about health consequences)*	76.9 (10)	47.1 (8)	29.8	72.7 (8)	41.2 (7)	31.5	NA
	… that patients are more likely to attend screening if a health professional prompts or encourages them to do so. *(9.1 Credible source)*	84.6 (11)	70.6 (12)	14	90.9 (10)	64.7 (11)	26.2	NA
**(vii) Give practices feedback…**	…On national or international uptake or targets *(2.2 Feedback on behaviour and 1.6 Discrepancy* *between current behaviour and goal)*	76.9 (10)	82.4 (14)	5.5	100 (11)	88.2 (15)	11.8	Motivating practice staff to make sure the person attends screening (HCPs)
	Use a trusted source to deliver feedback and messages (e.g. colleague) *9.1 Credible Source*	91.7 (11)	81.3 (13)	10.4	76.9 (10)	68.8 (11)	8.1	NA

People with diabetes = People with Diabetes, HCP= Health Care Professional, NA = Not Available e.g. there was no data/themes from the consensus meetings which could explain the agreement ratings, BCT = behaviour change techniques

***Concerns about intervention content***

**Figure 2.**
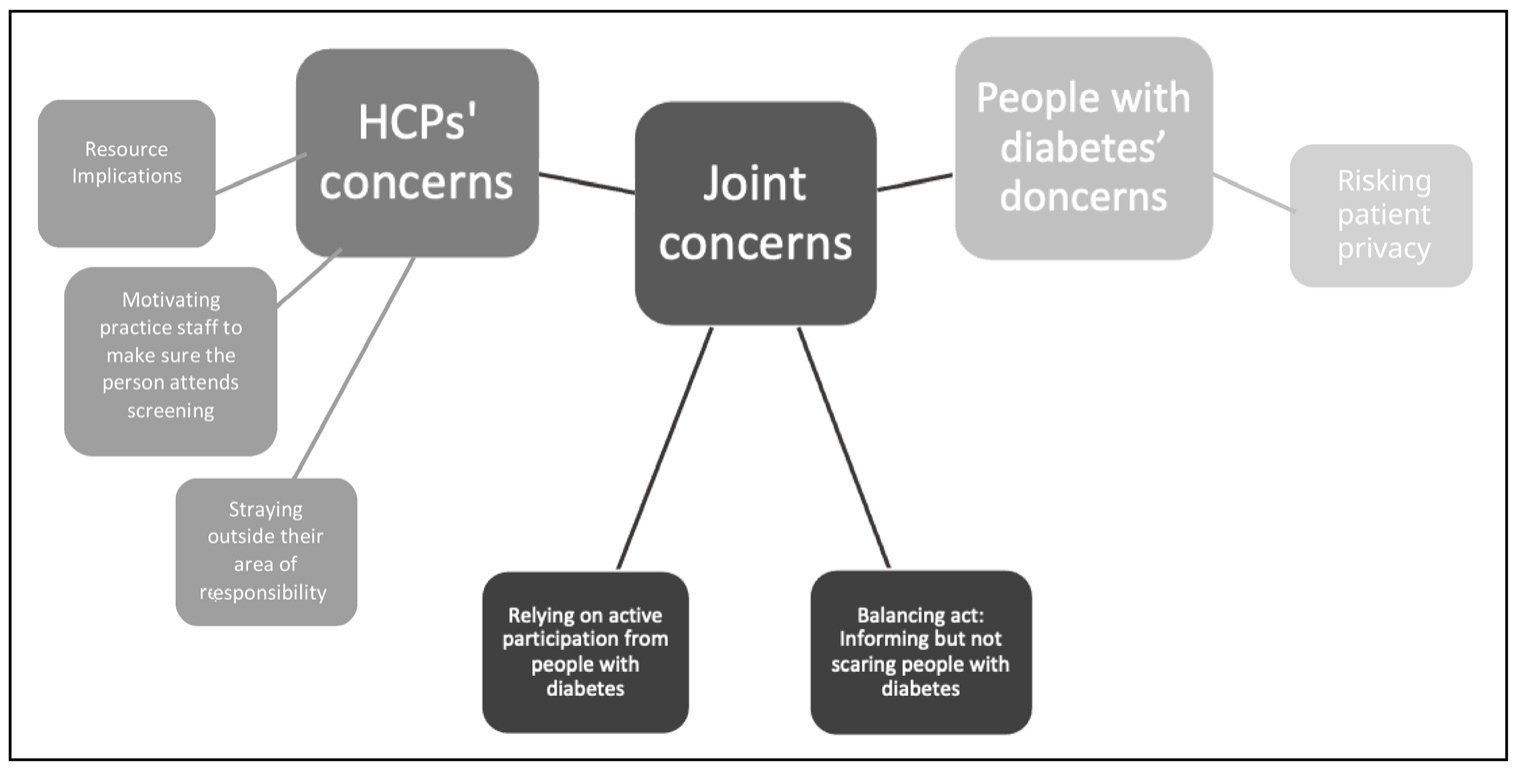
Concerns about intervention content organised by health care provider (HCP) concerns, joint concerns, or people with diabetes’ concerns.

Following integration of the survey results and qualitative feedback from the consensus meetings, themes related to the preference for and several main concerns about acceptable and feasible intervention content ( Figure 2). Table 1 presents where these relate to intervention proposals and whether it was a joint concern, or preference, of both people with diabetes *and* HCPs, HCPs *only* or people with diabetes *only*.

The results are organised according to the joint preference, joint concerns, HCP concerns and people with diabetes’ concerns. Examples of intervention proposals that relate to each area of concern are presented, along with the survey results and a short summary of participants’ feedback from the consensus groups.

***Joint preference***

-
*Some people with diabetes have a limited understanding of the need for and practicalities of screening*

Participants in all three meetings considered several intervention proposals to be acceptable and feasible because they believed some people with diabetes have a limited understanding of the screening process. In the survey, both people with diabetes and HCPs agreed the proposal to *use someone in the practice who would explain the difference between routine eye checks and the screening test* was acceptable (92.3% vs. 94.1%, respectively), though they differed in agreement with feasibility (75% vs. 94.1%, respectively). Data from the meetings provided no indication as to why people with diabetes rated feasibility lower than HCPs, however both groups flagged that there is confusion among some people with diabetes about the difference between routine eye tests and retinal screening. Participants in the combined meeting agreed that messages delivered to patients should outline the difference between routine eye tests and retinal screening and emphasise that damage can be asymptomatic to dispel the “false sense of security”. Similarly, participants in the people with diabetes only meeting thought messages should aim to increase patient understanding of the screening process. For example, highlighting the possible consequences of non-attendance and “alert you (people with diabetes) to the dangers involved”. Participants in the HCP only meeting agreed messages should emphasize that screening is free.

In the survey, less people with diabetes than HCPs agreed the proposal to arrange practical support was acceptable (66.7% vs 82.4% respectively), though less HCPs agreed it was feasible (58.3% vs HCPs 41.2%). Participants in the people with diabetes only meeting felt many people with diabetes are not aware of the need to organise transportation for after the screening procedure, and so messages should tell people they would need support rather than arranging it for them. HCPs had concerns about the feasibility of this proposal, which are discussed below under the concern *straying outside their area of responsibility*.

***Joint concerns***

-
*Relying on active participation from people with diabetes*

Some HCPs and people with diabetes had concerns about proposals which might rely on active participation from people with diabetes, for example, the proposal for *the person with diabetes to tick off a checklist when they have consented to/attended to screening*. In the survey, a larger proportion of people with diabetes than HCPs agreed providing a checklist would be acceptable (69.5% and 35.3%, respectively) and feasible (75% and 35.3%, respectively). In the people with diabetes only and combined meeting, some people with diabetes felt having a checklist would help people be “proactive” in the management of their diabetes, while others thought that this would put too much responsibility on the person who “might lose or forget it”. Some of those in the HCP only meeting thought that only motivated and engaged patients would use the checklist.

-
*Balancing Act: Informing but not scaring people with diabetes*

Participants from all three meetings were concerned about achieving the balance between communicating the risks of diabetic retinopathy while not scaring people when informing them about screening. This concern related to several proposals to use other people with diabetes or HCPs to deliver messages. In the survey, both HCPs and people with diabetes agreed it would be acceptable to use a message from someone who *has retinopathy and tells them it is important to go to screening before it is too late, there may be no symptoms and everyone with diabetes is at risk*. However, 83.3% of people with diabetes agreed it would be feasible compared to 64.7% of HCPs. Participants across all meetings believed that “scaremongering” or “shock tactics” would not encourage people to attend. Rather than “shock” people, messages should inform them of the “truth” about the possible consequences of non-attendance and be provided “by the right person, in the right way”. Both people with diabetes and HCPs agreed that the same message ( *tells them it is important to go to screening before it is too late, there may be no symptoms and everyone with diabetes is at risk*) when delivered by HCP rather than another person with diabetes would be acceptable (100% and 88.2%, respectively) and feasible (91.7% and 82.3%, respectively). Participants in the people with diabetes only meeting thought the GP would be the best person to deliver a message to attend screening as people “trust” their GP and are “much more inclined to listen to them”. HCPs in the HCP only and combined meeting had concerns that that delivering these messages during consultations would take a considerable amount of time.

***Health care professionals’ concerns***

-
*Resource implications*

Concerns about the resource implications of delivering intervention proposals including time, staff, and money, were raised throughout all three meetings. Resource concerns were often a reason for HCPs’ lesser agreement with proposals, especially those which aimed to encourage practice staff to ensure the person attends. Few people with diabetes and HCPs thought the proposal to *provide a new resource to the practice (e.g., researcher checks if person registered, consented and/or attended)* was feasible (53.8% vs 58.8%, respectively). While both agreed the proposal to *prompt practice to check the* (DRS) *register during consultation and register person if necessary* was acceptable, a slightly lower proportion of HCPs thought it was feasible (82.4% and 70.6%, respectively). They emphasized not having time for multiple prompts and reminders like letters or emails; “we absolutely don't have the time. We can't take anything on, it’s just beyond unbelievable.”

-
*Motivating practice staff to make sure the person attends screening*

HCPs in both meetings had concerns about proposals to tell practices about the benefits/consequences of their patients attending/not attending. This was reflected in the different proportions who agreed such was feasible (people with diabetes 90.9% vs HCP 70.6%, respectively). Some HCPs believed financial incentives might be best to motivate GPs to ensure their patients are registered and attend DRS. HCPs in the combined meeting suggested that once practices have a registration uptake at a particular level, they could receive financial remuneration and therefore be “incentivised to do it (register patients)”. There were also concerns about using feedback to motivate HCPs to encourage patients to attend, namely by providing practices with comparison numbers (% people attending in other practices/ nationally). This discussion arose around the proposal to *give feedback on national or international uptake or targets*. Some participants in the HCP only meeting felt “you would totally tap into [competitive] personalities” but there was a lack of consensus on this proposal in the combined meeting. Some participants in this meeting thought a comparator could be a useful motivator, whereas one GP noted that the differing demographic of patients across practices would make comparisons difficult. HCPs in both meetings argued that feedback needs to be specific and tailored to their practice and their patients, as national averages and practice comparisons are “totally useless” as they “cannot address that on a one-to-one level with a patient”.

-
*Straying outside their area of responsibility*

As previously mentioned, the proposal to arrange practical support like transportation was not considered feasible by people with diabetes nor HCPs (58.3% vs 41.2%, respectively). HCPs in the HCP only and combined meeting felt this proposal strayed outside of their area of responsibility, as they mostly interpreted it as having to arrange the transportation for the patient themselves, something they felt was “not their (HCP) problem” as patients “need to take ownership and responsibility”.

***People with diabetes’ concerns***

-
*Risking patient privacy*

Participants in the people with diabetes only meeting were concerned that some proposals threatened their privacy. For example, arranging practical or social support would make it difficult for those who wish to keep their diabetes private to do so. Both people with diabetes and HCPs thought the proposal to provide a new resource to the practice like a researcher was not feasible (53.8% and 58.8%, respectively). A few participants in the people with diabetes only meeting were concerned about privacy should someone within the practice other than their GP/PN have access to their information. Contrastingly, more people with diabetes than HCPs thought this proposal would be acceptable (83.3% vs 64.7% respectively). However, this may be explained by HCP concerns about resourcing this proposal.

### Comparing stakeholders’ suggestions for new intervention content

Participants in the people with diabetes only meeting made 26 suggestions for new intervention content, of which 7 were deemed feasible to incorporate into the final intervention (30%). Participants in the combined meeting also made 26 new suggestions, of which 3 were feasible (15%). Participants in the HCP only meeting made 32 new suggestions, of which 7 were feasible (22%). Table 2 shows the suggestions for new intervention content that were deemed feasible to incorporate. New suggestions were deemed unfeasible to incorporate into the intervention if they could not be implemented in the Irish general practice setting. For example, participants in all three meetings suggested that the reminder message should be delivered by professionals outside general practice, that the national screening programme could modify their processes to make it easier for people with diabetes to register and attend the service, and that national-level changes (e.g., media campaign to improve attendance, establishing a national diabetes register) should be introduced to increase screening attendance.

**Table 2.** Suggestions for new intervention content that were deemed feasible to incorporate into the intervention.

Suggestion	People with diabetes only meeting	Combined meeting	HCP only meeting	BCT	Incorporated into the final intervention
**Patient-level proposals**					
Visuals should not be gruesome	✓	-	✓	*n/a*	✓
Distinguish the difference between HBA1c and retinal screening	✓	✓	-	*5.1 Information about health consequences* *13.2 Framing/ reframing*	□
Outline that GP has noticed that the patient has not attended	✓	-	-	*2.2 Feedback on behaviour* *6.3 Information about others’ approval*	✓
GP should recommend that the patient talks to another patient at the practice	✓	-	-	*6.2 Social comparison* *6.3 Information about others approval*	□
Do not use scaremongering language	-	-	✓	*n/a*	✓
Personal story from a celebrity	-	-	✓	*9.1 Credible source* *6.2 Social comparison* *6.3 Information about others’ approval*	□
Provide a link to further information online	-	-	✓	*5.1 Information about health consequences*	□
Ask patients to attend as a favour to the practice to get their numbers up	-	-	✓	*6.2 Social Comparison* *13.2 Framing/ reframing*	□
Tell patients that they need to prioritise their eyes, emphasise how important they are compared to other things	-	-	✓	*5.1 Information about health consequences*	✓
Patients should be reminded to attend screening before they come to the practice to collect their next prescription as a ‘subtle threat’	-	-	✓	10.1 Material incentive (behaviour) * 14.2 Punishment *	□
**Practice-level proposals**					
One person at practice dedicated to reminding patients to attend screening	✓	✓/✗ ^1^	-	12.1 Restructuring physical environment *	✓
Have a chart at practice with the % numbers they want to achieve	✓	-	-	1.3 Goal setting * *12.5 Adding objects to the environment*	□
Inform practices that they can market themselves as a practice known for good diabetes care	✓	-	-	*5.3 Information about social and environmental consequences*	□
Practice staff should be shown how to use the GP software to check screening registration and attendance	-	✓	-	*12.1 Restructuring physical environment*	✓

^1^Conflicting opinions -Either participants in one small group agreed but participants in another small group disagreed with the recommendation or participants in one small group agreed but later in the discussion participants in the same small group disagreed with the recommendation.*BCT identified in the new suggestions that was not present in the intervention proposals outlined in the survey. Abbreviations: HCP = Health care professional, BCT = behaviour change techniques

New suggestions deemed feasible to incorporate into the intervention mapped to 12 BCTs in the taxonomy ( Table 2). There were four additional BCTs identified in the new suggestions that were not present in the intervention proposals outlined in the survey: *goal setting (outcome)*, *restructuring the physical environment*, *material incentive (behaviour)* and *punishment*. Additional information on the BCTs identified is provided in Supplementary File 7 in the *Extended data* ^18^.

### Comparing end users’ contributions to the final intervention

The final intervention included a practice briefing, audit and feedback with technical support, practice-endorsed reminders (delivered in person, by phone and letter) and an information leaflet targeting key attitudinal and knowledge barriers. The people with diabetes only meeting had 23/51 (45%) recommendations incorporated into the final intervention, of these 20 were feedback on the intervention proposals and three were new suggestions. The combined meeting had 19/49 (39%) recommendations incorporated into the final intervention, of these 17 were proposed and two were new suggestions. The HCP only meeting had 24/55 (44%) recommendations incorporated into the final intervention, of these 21 were proposed and three were new suggestions. Table 2 shows the new suggestions that were incorporated into the final intervention. All three meetings made new suggestions that were deemed feasible but not incorporated into the final intervention. These suggestions, along with the reasons for exclusion (based on the APEASE criteria), are outlined in Supplementary File 8 in the *Extended data* ^18^.

## Discussion

### Summary of main findings and links to existing literature

Although there is growing awareness in the literature that involving different intervention end-users in the development process may have a different impact on the final intervention developed ^8, 11, 27^, to our knowledge, this is the first study to examine and compare in detail the contributions of different intervention end-users as part of a consensus approach to inform intervention development.

There were three main findings. Firstly, people with diabetes and HCPs had both shared and unique opinions about the acceptability and feasibility of some aspects of the proposed intervention content. Some opinions were shared by both end-users and were echoed throughout all three consensus meetings, for example that there is a limited understanding of the screening process, or that we should balance informing people without scaring them when communicating about screening. However, HCPs also had unique concerns related to their role as healthcare providers, while people with diabetes had their own concerns about intervention proposals which might risk their privacy. Such differences suggest that while there is a common ground when it comes to preferences for and concerns about intervention content, there are some aspects of the intervention which may be a greater priority for different end-users. Secondly, participants in all three meetings made suggestions for new intervention content which mapped to BCTs that were not present in the proposed intervention content however, participants in the combined meeting made less feasible suggestions as they could not be implemented in the Irish general practice setting. Finally, participants in all three meetings made recommendations that were incorporated into the final intervention. However, participants in the combined meeting had fewer recommendations incorporated than the other two meetings.

In the meetings involving people with diabetes only and HCPs only, respective groups had different opinions about the delivery of messages to attend screening e.g., who should deliver the message, when the message should be delivered, and what the message should contain. Those in the meeting of people with diabetes only tended to base their recommendations on what would be most acceptable to the person with diabetes. In contrast, participants in the HCP only meeting focused more on what was feasible from a resource perspective. These concerns are consistent with reports of increased workload and staff burnout in Irish general practice ^28, 29^. In addition, some HCPs perceived that certain intervention proposals would involve straying outside their area of responsibility. They tended to disagree with proposals which they equated to an extra job or responsibility, understandable given the increasing responsibilities in general practice for chronic disease management ^30^. Future intervention developers should consider these different perspectives of respective end-users so that they may involve them in the development process in the most effective way.

On the other hand, participants in this study also had joint preferences for intervention content. Both HCPs and people with diabetes were conscious that while it was important to outline the seriousness of retinopathy, there is a need to strike a balance between informing but not scaring people about the screening process and potential disease consequences from non-attendance. This aligns with the body of literature on the use, or avoidance, of fear appeals to encourage preventative health behaviours, evidence which has demonstrated that providing information about possible negative consequences may prompt defensive responses ^31^. For instance, one US study found that avoidance of cancer risk information was associated with lower participation in colorectal cancer screening ^32^. During the consensus meetings, people with diabetes and HCPs had concerns about intervention content which might scare or frighten people, such as having a message delivered by someone who is visually impaired or prompting the person to feel regret. Intervention developers should select behaviour change techniques that promote adaptive, rather than maladaptive behaviour, as suggested by a qualitative study of fear appeals as a method in behaviour change interventions ^33^. These joint contributions by participants in our study offer a useful perspective to intervention developers about how end-users will receive communication, but also demonstrates there are instances where end-users can share priorities for intervention content. 

Our findings indicate that end-user groups’ contributions to the intervention development process can differ based on whether they are involved separately or simultaneously. Participants in the combined meeting of people with diabetes and HCPs made fewer feasible suggestions for new intervention content and fewer recommendations from this meeting were incorporated into the final intervention. This suggests their contributions may have been influenced by group composition. Our previous analysis of participants’ experiences of taking part in the consensus meetings found that, although members of the combined meeting appeared to work together, during follow-up data collection both end-user groups held different views about what intervention proposals would and would not work ^13^. Both HCPs and people with diabetes that participated in the combined meeting were uncomfortable with asserting what the other end-user group should or should not do. To fill this void, participants went off task and made suggestions that outside the scope of an intervention intended for primary care ^13^. This current study alongside our previous analysis suggests that it may be useful to involve each end-user group, those who will deliver the intervention and the intended target population, separately rather than simultaneously in a consensus process to inform intervention development. When involving different end-users together in a consensus process, researchers should also consider facilitating these groups differently, paying special attention to acknowledge potentially unique views while also reaching consensus. Previous research has recognised the potential complexity of multi-stakeholder involvement, highlighting the need to manage group interactions, potential power imbalances and synthesising the views of different groups ^34^. One approach which might have been useful in the context of our research and could be relevant to future work in this field, would be hold the separate stakeholder groups first to allow for independent discussion and feedback, followed by a combined group in which consolidated feedback may be compared and discussed. 

### Strengths and Limitations

This study has several strengths including the use of a mixed methods, explanatory sequential design. Consensus meeting data supported the quantitative analysis by providing explanations, where available, for different participant ratings provided in the survey. By integrating the two, we aimed to draw out new findings beyond the information gained from the separate results ^35^. Fetters *et al.* have reported that such qualitative methods are often applied in order to explore reasons why a phenomenon occurs or to describe the nature of an individual's experience ^17^. The involvement of PPI contributors is a further strength of this research. A PPI partner (GF) was involved in the SWAT throughout the duration of the study and is a named co-author on this publication. A separate PPI group were involved in the development of the materials sent to participants prior to the consensus meetings. Supplementary files 3.2. and 3.3 in the *Extended data* ^18^ show how the study invitation letter and survey were improved as result of PPI feedback. These improvements helped to ensure that materials were more accessible and acceptable to participants.

This study includes a number of limitations. Firstly, as this was a SWAT, the primary aim of the consensus meetings was to review and discuss proposals for intervention content for the host trial and not to explicitly compare end-user contributions ^15^. While the semi-structured approach of the meetings allowed participants to discuss proposed intervention content and generate new ideas for such content, it made it difficult to compare end-user contributions as the content and nature of the discussions varied across meetings. For example, some groups did not discuss certain survey ratings and intervention proposals, and some groups discussed particular proposals in more detail than others. This meant that explanations for survey ratings are not present in qualitative form consistently for all intervention proposals. Adopting a more structured approach, for example the nominal group technique or Delphi method ^36^, during the consensus meetings may have made it easier to compare views on all proposals across groups.

An additional limitation is the absence of some key end-users from the consensus meetings. There were no people with type 2 diabetes available to participate in the combined meeting. Despite using a range of strategies to recruit a representative sample of people with diabetes, we encountered issues with participant availability when arranging the combined meeting. Existing research has established that people with type 1 and type 2 diabetes have different experiences when managing their condition and engaging with HCPs and health services ^37– 39^. Therefore, the involvement of people with type 2 diabetes in the combined meeting could have potentially changed the nature of the discussion and led to different recommendations. There was also a lack of involvement of practice administrators in the consensus meeting. Participants in the HCP only meeting suggested that practice administrators would be best placed to deliver the intervention. Involving them in the consensus meetings may have led to different recommendations as they play a key role in undertaking clerical duties to support delivery of care, and as gatekeepers, help to preserve boundaries of organisation and controlling access to the practice ^40^. However, the literature finds they are often overlooked by policymakers, undervalued by GPs and patients and excluded from research ^40^. Future research in general practice should consider involving practice administrators to ensure that all user voices are heard.

A final limitation was the lack of capture of non-verbal cues such as when participants nod in agreement or disagreement. As this SWAT looked to examine and compare agreement with proposed intervention content, such non-verbal data may have been useful. While non-verbal cues can offer rich data ^41^ and we may have been able to capture this through video recording of the meeting, it has also been found that the use of video-recording equipment during focus groups can inhibit participants’ interaction ^42^.

### Implications

The results of this SWAT informed the development of the IDEAs intervention which has been tested as part of a pilot cluster randomised trial with a view to progressing to a definitive trial ^14^. Involving end-users in decisions about planning and conducting health research, policy and services is gaining increasing momentum and as such, PPI is now required by many health research funders, journals, and research ethics committees ^43, 44^. However, evidence on the impact of PPI is largely based on anecdotal reflections from researchers and members of the public which are descriptive and selective ^45^. Numerous studies have called for planned and methodologically rigorous research to evaluate the impact of PPI on the research process ^46– 48^. In this study, the lines between research participation and involvement were blurred, as is often the case with PPI ^49^. Although people with diabetes were research participants in the consensus meetings, their role in the consensus meeting was to discuss and make decisions about the intervention content and mode of delivery which could be viewed as PPI ^50, 51^. This SWAT provides evidence on the contribution of different end-users to the intervention development process and how different end-users can have different priorities for intervention content.

The results of this study, coupled with the results of our analysis of participants’ experiences of taking part in the three separate meetings to inform intervention development ^13^, suggest that it may potentially be more acceptable and useful to involve patients/members of the public and HCPs separately when conducting PPI activities. When involving stakeholders together in PPI activities, alternative approaches to facilitation may need to be considered. Furthermore, as the process and impact of PPI is heavily dependent on the context in which it is being conducted, further research exploring the experiences and contributions of different end-users is needed, including an exploration of different facilitation models. This would enable all individuals interested in involving patients and members of the public in health research, policy, planning and development of health care to design and conduct more appropriate and effective user involvement ^8, 52^.

## Conclusion

UK Medical Research Council guidance on the development and evaluation of complex interventions states that interventions should be developed with user involvement, drawing on existing evidence and appropriate theory ^1^. However, there is limited evidence on what different intervention users contribute to the intervention development process and whether their contributions differ according to group composition. Our findings show that preferences and priorities for intervention content can differ across end-user groups, and that suggestions and recommendations for intervention content and design may also vary depending on whether users are involved simultaneously or separately. Considering these findings, attention should be paid to how end-users are involved in intervention development processes. This will stand to help researchers to design and conduct more appropriate user involvement, which in turn, could potentially improve intervention fit with the end-user’s perceived needs.

## Data availability

### Underlying data

The consensus meeting data are not publicly available due to limitations based on the ethical approval received and participant consent. Participants of the consensus process were not asked for their consent to store their data in a public repository. Participants consented to their anonymised data being made available for further collaborative research purposes outside of the current study upon reasonable request from the corresponding author and provision of a written proposal to the Principal Investigator (Dr Sheena McHugh, S.McHugh@ucc.ie).

Open Science Framework: What and how do different stakeholders contribute to intervention development? A mixed methods study. https://doi.org/10.17605/OSF.IO/NJS9Y ^18^.

The project contains the following underlying data:

- Quantitative_Data_Survey_Results_anon.xlsx
- Quantitative_Data_Codebook.docx

Data are available under the terms of the Creative Commons CC0 1.0 Universal (CC0 1.0) Public Domain Dedication License. 

### Extended data

Open Science Framework: What and how do different stakeholders contribute to intervention development? A mixed methods study. https://doi.org/10.17605/OSF.IO/NJS9Y ^18^.

This project contains the following extended data:

- Supplementary File 1. Recruitment Survey
- Supplementary File 2. Evidence Summary
- Supplementary File 3. Self-completion survey of intervention components
- Supplementary File 4. BCTs operationalised across the survey intervention proposals
- Supplementary File 5. Facilitator Guide
- Supplementary File 6. Mapping new suggestions to the BCT Taxonomy
- Supplementary File 7. BCTs identified from new suggestions that were deemed feasible to incorporate into the intervention.docx
- Supplementary File 8. New suggestions that were deemed feasible to incorporate but were not incorporated into the final intervention

Data are available under the terms of the Creative Commons CC0 1.0 Universal (CC0 1.0) Public Domain Dedication License. 
