# Supplementary material for: What and how do different stakeholders contribute to intervention development? A mixed methods study
Source: HRB Open Res. 2023 Feb 8;5:35. Originally published 2022 May 10. [Version 2] doi: 10.12688/hrbopenres.13544.2 (PMC9989546; doi:10.12688/hrbopenres.13544.2)
Supplement: Peer review history

# Peer review: Reviewer response for version 2

**DOI:** 10.21956/hrbopenres.14971.r33435

**Published:** 2023-03-06

**Authors:** Jo River

**Affiliations:** 1 Faculty of Health, University of Technology Sydney, Sydney, NSW, Australia

Thank you for the opportunity to re-review this paper. I am satisfied that the authors have attended to all comments. I found the responses thoughtful and comprehensive and would like to congratulate the authors on the publication.

 I am happy to support the paper to be indexed.

Is the work clearly and accurately presented and does it cite the current literature?

Yes

If applicable, is the statistical analysis and its interpretation appropriate?

Partly

Are all the source data underlying the results available to ensure full reproducibility?

Partly

Is the study design appropriate and is the work technically sound?

Yes

Are the conclusions drawn adequately supported by the results?

Partly

Are sufficient details of methods and analysis provided to allow replication by others?

Yes

Reviewer Expertise:

NA

I confirm that I have read this submission and believe that I have an appropriate level of expertise to confirm that it is of an acceptable scientific standard.

# Peer review: Reviewer response for version 1

**DOI:** 10.21956/hrbopenres.14784.r33196

**Published:** 2022-12-15

**Authors:** Shanna C. Trenaman

**Affiliations:** 1 Nova Scotia Health, Halifax, NS, Canada; 2 College of Pharmacy, Faculty of Health, Dalhousie University, Halifax, NS, Canada

This was a wonderful paper outlining stakeholder engagement for patient representatives and healthcare provider representatives in a trial implementing a intervention to improve retinal screening among people living with diabetes. I find it difficult to find problems with this paper. The introduction sets the stage, the methods outline the parent trial and goals of the study here. The mixed methods complement each other and integrate very well compared to many mixed methods trials which are often comprised of quantitative methods and qualitative methods which exist in stand alone different components. I was very much impressed with this work.

Is the work clearly and accurately presented and does it cite the current literature?

Yes

If applicable, is the statistical analysis and its interpretation appropriate?

Yes

Are all the source data underlying the results available to ensure full reproducibility?

Partly

Is the study design appropriate and is the work technically sound?

Yes

Are the conclusions drawn adequately supported by the results?

Yes

Are sufficient details of methods and analysis provided to allow replication by others?

Yes

Reviewer Expertise:

Geriatric Medicine, Pharmacy, Deprescribing, Dementia, Epidemiology

I confirm that I have read this submission and believe that I have an appropriate level of expertise to confirm that it is of an acceptable scientific standard.

# Peer review: Reviewer response for version 1

**DOI:** 10.21956/hrbopenres.14784.r32436

**Published:** 2022-08-04

**Authors:** Jo River

**Affiliations:** 1 Faculty of Health, University of Technology Sydney, Sydney, NSW, Australia

Thank you for the opportunity to review this manuscript which explores the important issue of how ‘end-users’ are involved in health intervention development. It is a very well-written paper and an interesting topic. The background, methods, and findings are well described. I do, however, have some suggestions for the conceptual development of the paper as well as one or two small suggestions for revisions.

 In terms of conceptual development, I found the positioning of people with diabetes and healthcare professions (HCP) as different kinds of ‘end-users’ somewhat problematic. While HCP certainly have an important stake in discussions about healthcare interventions, people with diabetes would surely be, as Daya (2020) ^1^ has previously noted, the ‘key players that everyone else has a stake in’. I wonder how this positioning might have influenced the facilitation process, e.g., how a lack of group clarity around this might make it hard for people with diabetes to articulate ideas in combined meetings. Further to this, the researchers themselves appeared to be an invisible power in the decision-making process, with the power to determine what ideas were feasible/not feasible to include in the intervention outside of intervention development meetings. I wonder what might be different if people with diabetes and HCP had they had the opportunity to engage more deliberately in a dialogue around these decisions and how this might have shaped the final intervention. The researchers also note that a ‘male professor’ facilitated meetings, but it is unclear who this person was in relation to the research team, or how this might have influenced the process. For example, what might be different if a person with diabetes on the research team had co-facilitated? How might this support people in the combined groups to talk through conflicting views?

 In terms of methodology, I do not think that the involvement of PPI was ‘blurred’ in the study (p.16). I think it is important to clarify the distinction between ‘participants in research’ and ‘PPI research collaborators or co-researchers’. People with diabetes who were participants in the research study were involved in developing the intervention and would not constitute PPI research collaborators or co-researchers. While study participants had some decision-making power over the intervention, they did not have any decision-making power over the research strategy or interpretation of data. This is distinct from PPI research involvement, which in this study appears to be one person with diabetes (GF), who contributed to the research strategy including initial discussions that informed the grant application, development of recruitment materials, recruitment of people with diabetes to the study, and review of a draft publication. It is also important not to overstate PPI research involvement in this study. While it is certainly valuable to have input from one person with diabetes in the research project, ideally people with lived experience would be involved in equal numbers throughout all stages of the research process to ensure research priorities and interpretations are relevant and resonant to those most impacted by research-informed policy and services.

 In terms of interpretation of findings, while it is interesting to consider how separating or bringing people together might influence intervention development, I think findings need to be interpreted more cautiously. Participant numbers are small, and many factors may have contributed to the number of suggestions made or ideas incorporated into the final intervention – not the least of which is the researchers’ power to make this final decision.

 Finally, some small issues relate to the term ‘diabetes only’ in the abstract. I think it would be best that groups are not named by an illness, and person first language would be preferable, e.g., ‘People with diabetes, or PWD only’ meeting would seem a better description, particularly as the term ‘HCP only’ meeting is used. It would also be good to use the full term for 'BCT' in Table 2 to make this more convenient for the reader.

 I hope that this review is useful to the research team and support them in furthering the important work they are doing in determining best practice approaches to the development of interventions with people with lived experience and other key stakeholder groups.

Is the work clearly and accurately presented and does it cite the current literature?

Yes

If applicable, is the statistical analysis and its interpretation appropriate?

Partly

Are all the source data underlying the results available to ensure full reproducibility?

Partly

Is the study design appropriate and is the work technically sound?

Yes

Are the conclusions drawn adequately supported by the results?

Partly

Are sufficient details of methods and analysis provided to allow replication by others?

Yes

Reviewer Expertise:

Co-design, mental health, drug and alcohol research

I confirm that I have read this submission and believe that I have an appropriate level of expertise to confirm that it is of an acceptable scientific standard, however I have significant reservations, as outlined above.

## References

[1] : The Participation Ladder: A Consumer/Survivor Lens.2020; Reference source

# Response

**Published:** 2023-01-31

**Authors:** Lauren O Mahony

**Affiliations:** University College Cork, Ireland

Thank you for taking the time to provide a very useful and in-depth review of our research manuscript. We have addressed the suggestions and comments in our revised manuscript.

 1) In terms of the positioning of people with diabetes and healthcare professions (HCP) as different kinds of ‘end-users’ somewhat problematic: This is a valid point, and one which we have discussed amongst the research team. We accept that in most interventions, patients or service users should be considered ‘key players that everyone else has a stake in’ , however, this current intervention was a multilevel intervention which targeted both people with diabetes and healthcare professionals,  that is, it had components that targeted people with diabetes (i.e., personal testimonials, reminders, information provision etc.) and professionals working in general practice (i.e., audit, feedback, electronic prompts etc.). Therefore, during the consensus meetings, people with diabetes and healthcare professionals were asked to discuss patient- level and practice-level components that were to be implemented as part of the intervention. It was made clear at the outset of the meetings that the focus was both people with diabetes and HCPs. We agree that this positioning likely influenced how PWD (and HCPs) contributed during the combined meeting. This is reflected in the discussion on our previous analysis of both PWD and HCP experiences of taking part in the consensus meetings in lines 467-481. We also agree with your suggestion that the researchers were an ‘invisible power’ in the decision-making process, who influenced the final intervention and have added a paragraph to the discussion section to address this in line 538-549.

 2) Regarding the ‘male professor’ who facilitated meetings: We have added additional details to the methods section to clarify the relationship between the male facilitator and the research team in lines 122-125. We have also added sentences to the discussion to consider how involving a person with diabetes as a co-facilitator may have impacted the process; lines 482-489. 

 3) In terms of PPI methodology, we have taken your point on board and agree with your suggestion to clarify the distinction between research participants and PPI contributors. We have re-written the sentences in question as following in lines 582-586. In terms of PPI involvement in this study, GF was involved throughout the research process as you have correctly pointed out. We also consulted with an existing PPI group on the design of the consensus meeting invitation letter, evidence summary and self- completion survey. We have added further information to the methods section to give a more accurate depiction of the role of PPI in this study; lines 221-232. 

 4) In terms of cautious interpretation of findings, we have now included further description in the i) Discussion (lines 503-512) and ii) Study Implications (lines 588-591). 

 5) Thank you for pointing out the typo in the abstract. This is most certainly an error, as we fully appreciate the importance of using terminology which ensures individuals are not identified by an illness and have endeavoured to use appropriate language throughout the study. We have now amended this to say ‘PWD only’ in the abstract methods. We have also amended Table 2 as per your suggestion.
